# Connexin32 plays a crucial role in ROS-mediated endoplasmic reticulum stress apoptosis signaling pathway in ischemia reperfusion-induced acute kidney injury

**DOI:** 10.1186/s12967-018-1493-8

**Published:** 2018-05-04

**Authors:** Yu Gu, Fei Huang, Yanling Wang, Chaojin Chen, Shan Wu, Shaoli Zhou, Ziqing Hei, Dongdong Yuan

**Affiliations:** Department of Anesthesiology, The Third Affiliated Hospital of Sun Yat-sen University, No. 600 Tianhe Road, Guangzhou, 510630 Guangdong Province China

**Keywords:** Acute kidney injury, Connexin32, Reactive oxygen species, Endoplasmic reticulum stress

## Abstract

**Background:**

Ischemia–reperfusion (I/R)-induced acute kidney injury (AKI) not only prolongs the length of hospital stay, but also seriously affects the patient’s survival rate. Although our previous investigation has verified that reactive oxygen species (ROS) transferred through gap junction composed of connexin32 (Cx32) contributed to AKI, its underlying mechanisms were not fully understood and viable preventive or therapeutic regimens were still lacking. Among various mechanisms involved in organs I/R-induced injuries, endoplasmic reticulum stress (ERS)-related apoptosis is currently considered to be an important participant. Thus, in present study, we focused on the underlying mechanisms of I/R-induced AKI, and postulated that Cx32 mediated ROS/ERS/apoptosis signal pathway activation played an important part in I/R-induced AKI.

**Methods:**

We established renal I/R models with Cx32^+/+^ and Cx32^−/−^ mice, which underwent double kidneys clamping and recanalization. ROS scavenger (*N*-acetylcysteine, NAC) and ERS inhibitors (4-phenyl butyric acid, 4-PBA, and tauroursodeoxycholic acid, TUDCA) were used to decrease the content of ROS and attenuate ERS activation, respectively.

**Results:**

Renal damage was progressively exacerbated in a time-dependent manner at the reperfusion stage, that was consistent with the alternation of ERS activation, including glucose regulated protein 78 (BiP/GRP78), X box-binding protein1, and C/EBP homologous protein expression. TUDCA or 4-PBA application attenuated I/R-induced ERS activation and protected against renal tubular epithelial cells apoptosis and renal damage. Cx32 deficiency decreased ROS generation and distribution between the neighboring cells, which attenuated I/R-induced ERS activation, and improved cell apoptosis and renal damage.

**Conclusion:**

Cx32 mediated ROS/ERS/apoptosis signal pathway activation played an important part in I/R-induced AKI. Cx32 deficiency, ROS elimination, and ERS inhibition all could protect against I/R-induced AKI.

**Electronic supplementary material:**

The online version of this article (10.1186/s12967-018-1493-8) contains supplementary material, which is available to authorized users.

## Background

AKI is a significant clinical complication with high morbidity and mortality, which is closely associated with prolonged hospital stays and increased hospitalization costs [1, 2]. It has been reported that the fatality rate of severe AKI exceeded 50% [3]. In a large amount of clinical settings, such as renal artery reconstruction, cardiac bypass surgery, partial nephrectomy, shock, and kidney transplantation, I/R-induced renal damage has been considered to be one of the most important causes that contribute to AKI [4, 5]. Usually, the clinical outcome of AKI is closely related to the severity of I/R-induced injury. Severe and sustained injury could lead to chronic kidney diseases and even end stage kidney diseases, which need dialysis or even renal transplantation [5]. Although, many potential mechanisms of I/R-induced AKI have been explored, viable preventive or therapeutic regimens are still lacking.

The etiology and pathogenesis of renal I/R injury is diverse and complex, however, its underlying mechanism is not understood very well. Connexin and its composed gap junction channel play a vital role in maintaining homeostasis and normal renal function. In our previous study, we had demonstrated that Cx32 inhibition might protect against liver transplantation-induced AKI, however, its effect on I/R-induced AKI had not been reported [6]. This is one of the key problems to be solved in our research. Connexins, a big family of transmembrane proteins, express in almost all human organs and tissues, exerting different pathological and physiological functions according to their characteristics [7]. Up to now, 21 isoforms have been isolated and been named by their own molecular weight, such as Cx32, Cx43, and Cx37 [8, 9]. There have been mainly nine isoforms of connexin protein found in kidney. Among them, Cx32 is abundantly expressed in the renal tubular system, especially in proximal tubules, which plays a key role in maintaining and regulating the function of renal tubules [6, 10]. Six connexins compose a hemichannel, the intact gap junction (GJ) channel is formed when one hemichannel docks with a second in an neighboring cell. Gap junctions allow small molecules (including calcium, trisphosphate, glutathione, ROS, cyclic guanosine monophosphate, cyclic adenosine monophosphate, etc.) transfer between neighboring cells, regulating the direct cell-to-cell signaling transfer [6, 11, 12]. This kind of signaling transfer is not only important for maintaining normal physiological functions, but also involves in the development of many diseases. ROS is one of the few signals that could be transferred through GJ. Our previous study has indicated that in renal tissues or renal tubular epithelial cells, the changes of ROS content and distribution are controlled by GJ composed of Cx32, but the downstream signaling pathways mediated by ROS have not been determined [6]. Under normal conditions, the level of ROS in tissues or cells is very low; however, it could be increased dramatically when tissues or cells are exposed to strong stress, which leading to oxidative stress, disruption of redox signaling pathway and apoptosis [13]. We found that ERS activation was closely linked to the increase of cells apoptosis and the exacerbation of tissues lesion after I/R injury [14].

ER is an organelle mainly responsible for the integration, fold, assembly and conveyance of protein in the cells [15]. It is sensitive to intracellular stress, can initiate/regulate adaptive responses, and also integrate cell apoptotic signals [16, 17]. When ER is disturbed by various stimuli, such as hypoxia, I/R, Ca^2+^ overload and ROS, its homeostasis is perturbed, causing numerous unfolded or misfolded proteins aggregated, motivating unfolded protein response (UPR), and resulting in the activation of ERS [18, 19]. Sustaining/overwhelming ERS converts pro-survival effects of adaptive UPR to pro-apoptotic death program [20]. So, when cells experience rigorous ERS, unable to restore ER lesion, the apoptotic pathways were activated [3]. Three kinds of ERS related proteins have been explored in present study: glucose regulated protein 78 (BiP/GRP78) is an ERS-related chaperone and master regulator of the UPR. It usually combines with three ER transmembrane receptors, and acts as signal transducers, including activated transcription factor 6 (ATF6), inositol requiring enzyme (IRE1), protein kinase-like ER kinase (PERK) in ER homeostatic conditions. In contrast, when ERS is activated, GRP78 dissociates from receptors and initiates related signaling pathways to reduce protein production and improve protein folding to repair the ordinary function of ER [21, 22]. X box-binding protein 1 (XBP1) is another ERS marker. The activation of XBP1 is also beneficial to restore ER homeostasis through degradation of misfolded proteins, thereby protect against cell damage, but the continuous increase of ERS will reverse this protective effect and activate pro-apoptotic pathway [23]. C/EBP homologous protein (CHOP) is a vital transcription factor facilitating ERS-initiated apoptosis, the level of which retains in a low level under normal circumstances, while sustained stress might exacerbate ERS and further increase CHOP expression, ultimately resulting in cell apoptosis and tissues damage [3].

Based on the facts mentioned above, we presume that the functional status of GJ composed of Cx32 influences the quantity and the distribution of ROS induced by I/R, which might further interfere ERS activation, and thus determines the outcome of AKI. So, we speculate that special knockout Cx32 gene, abundantly expressing in kidneys, might attenuate Cx32 GJ function, which decreases ROS production and delivery, thus further restrain the ERS-associated apoptosis pathway and moderate renal I/R injury. The in-depth understanding of these relevant factors in present study would help us to develop potential and feasible therapeutic targets for renal I/R-induced AKI.

## Methods

### Animals and treatments

Experimental protocols and implement were approved by the Institutional Animal and Use Committee of Sun Yat-Sen University. Animal care followed Guidelines of Sun Yat-Sen University for Animal Experimentation. Male C57BL/6 mice, 8–10 weeks old, weighing 20–25 g, were adopted for our experiments. Cx32^+/−^ mice purchased from European Mouse Mutant Archive (ID: 00243, Italy) on a C57BL/6 background, were used to breed Cx32^−/−^ and Cx32^+/+^ littermates. Polymerase chain reaction (PCR) was preformed to examine genotyping using tail snip extracted genomic DNA, as previously described [24]. All mice were housed with 12 h light/dark cycles at room temperature, and given free access to unlimited water and standard laboratory diet.

At the first intervention model establishment research, the Cx32^+/+^ mice were randomly assigned into 5 parallel groups (n = 6 per group) using a random number table taking into consideration of the weight of the mice. These include Sham operated, after I/R 8-, 16-, 24-, and 48-h groups. Subsequent studies were performed using the model of 24-h after I/R. Two well known ERS inhibitors, 4-PBA (100 mg/kg, i.p., 1-h before renal ischemia, Sigma-Aldrich, USA) or TUDCA (300 mg/kg, i.p., 1-h before renal ischemia, Sigma-Aldrich, USA), were used to decrease ERS activation. The Cx32^+/+^ mice were then randomly assigned to 6 groups (n = 6 per group) as following: Sham, I/R, Sham + (4-PBA), I/R + (4-PBA), Sham + TUDCA, and I/R + TUDCA groups. Furthermore, ROS scavenger, NAC (200 mg/kg, i.p., 1-h before renal ischemia, Sigma-Aldrich, USA), was used to explore the effectiveness of ROS on the activation of ERS and renal injury. The Cx32^+/+^ mice were randomly assigned to 4 groups (n = 6 per group), including Sham, I/R, Sham + NAC, and I/R + NAC groups. Finally, Cx32^−/−^ mice were used to explore effects of Cx32 on ROS, ERS and renal I/R injury. Cx32^−/−^ and Cx32^+/+^ mice were randomly assigned to 4 groups (n = 6 per group): (Cx32^+/+^) + Sham, (Cx32^+/+^) + I/R, (Cx32^−/−^) + Sham and (Cx32^−/−^) + I/R groups.

Renal I/R models were established as follows [3]: after abdominal incision, bilateral renal pedicles were occluded with a traumatic microvascular clamp for 45 min, then removed the clamp and restored blood flow. Mice in Sham group were undergoing the same procedures as I/R without clamping of renal pedicles. A heating pad was adopted to maintain body temperature stably at 37 °C during surgery.

Blood samples were collected at 8, 12, 24 and 48 h after reperfusion, then mice were sacrificed and the kidneys were obtained for further examinations.

#### Assessment of renal histopathological injury and renal function

Renal tissue samples obtained from mice were fixed in 4% paraformaldehyde, then embedded in paraffin and sliced into 4 μm sections. Hematoxylin and eosin (H&E) staining is used to evaluate renal histopathological injury. The histopathological score was estimated by an experienced pathologist who was blinded to the treated groups. The severity of renal tubules injuries were defined with 5 grades (0–4): 0, no obviously visible injury; 1, injury less than 25%; 2, injury about 25–50%; 3, injury about 50–75%; and 4, injury more than 75% [20]. The blood samples were collected from orbit and serum blood urea nitrogen (BUN) and creatinine (Cre) were measured with an automatic biochemistry analyzer (Epoch Chemray 240, Shenzhen, China) to assess renal function.

#### Western blotting analysis

Western blotting analysis follows the standard procedures as previously described [25]. Primary antibodies using as follows: anti-GRP78 (1:1000, Abcam, USA), anti-XBP1 (1:500, Abcam, USA), anti-CHOP (1:500, Cell Signaling Technology, Inc, USA) and anti-Cx32 (1:1000, Sigma-Aldrich, USA). Then, secondary antibody (Millipore, USA) and anti-GAPDH (Cell Signaling Technology, Inc, USA) were used at 1:5000. Image J scanning software was used to scan the image and the data were described as relative values to Sham values.

#### TUNEL assay

Kidney sections of mice from each group were TUNEL-stained using the In Situ Cell Death Detection Kit, POD (Roche Diagnostics GmbH, Germany) complying with the manufacturer’s instructions. Under optical microscope (400× magnification), the nucleus of TUNEL-positive cells were stained with brown, the number of TUNEL-positive cells in 10 sections from 6 different mice of each group were observed [25].

#### Immunohistochemistry and immunofluorescence

Renal tissue slides were de-waxed with xylene and dewatered with ethanol, then were microwave-heated to antigen retrieval in citrate buffer for 30 min. Nonspecific sites were blocked in goat serum. Next, sections incubated with anti-GRP78 (1:1000, Abcam, USA), anti-XBP1 (1:200, Abcam, USA), anti-CHOP (1:3000, Cell Signaling Technology, Inc, USA & 1:100, Abcam, USA) at 4 °C overnight, and then incubated with secondary antibody (1:500, Life technologies, USA) at 37 °C for 30 min. Ten fields of each section were chosen randomly to observe positively stained areas [26].

#### Tissue ROS assay

ROS generation in renal tissue frozen section was estimated by dihydroethidium (DHE) staining as described [25]. Briefly, frozen tissue sections were incubated in DHE (10 μM, Sigma-Aldrich, USA) at 37 °C for 30 min and then washed three times with PBS. The sections were sealed with reagent that resist fluorescence quench. Images observed and grabbed using EVOS FL fluorescence microscope (EVOS FL, Life Technology).

#### Statistical analysis

Data are presented as mean ± SEM. Statistical comparisons among groups were performed with SPSS 13.0 (SPSS Inc., Chicago, IL, USA) using one-way ANOVA following Tukey’s post hoc test. Each experiment was repeated at least three times. p < 0.05 was considered as statistically significant.

## Results

### Changes of I/R-induced renal injury and cell apoptosis were consistent with ERS activation

**Fig. 1:**
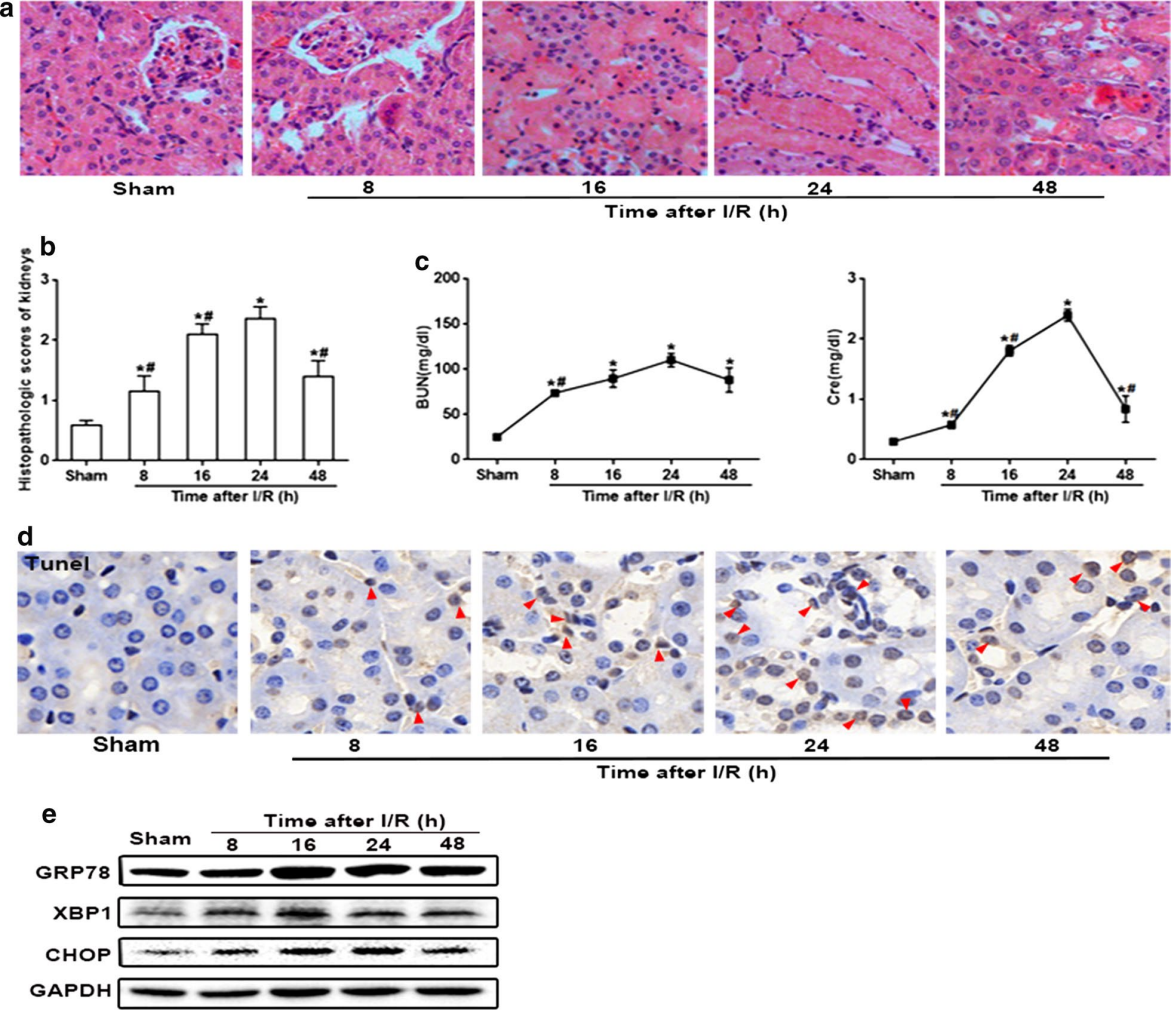
I/R-induced renal damage and renal tubular epithelial cells apoptosis were coincident with ERS activation. Samples of kidneys were obtained and evaluated after 45 min ischemia followed by different reperfusion times. **a** Representative light microscopy images of H&E-stained sections from every group (magnification ×200). **b** The graph shows histopathological score of kidneys in every group, n = 6, *p < 0.05 vs Sham group; ^#^p < 0.05 vs 24 h after I/R group. Data are the mean values ± SEM. **c** Representative serum concentrations of BUN and Cre at each time point, n = 6, *p < 0.05 vs Sham; ^#^p < 0.05 vs 24 h after I/R group. Data are the mean values ± SEM. **d** Representative TUNEL apoptosis assay images of kidney tissues. Brown dots were deemed positive apoptosis cells (magnification ×400; red arrow). **e** Renal I/R-induced ERS-related proteins (GRP78, XBP1 and CHOP) expression in renal tissues, which examined by western blotting analysis. GAPDH was used as the loading control and for band density normalization

Dynamic renal injuries were observed at different time points after renal I/R, which got to the peak at 24 h after renal I/R, manifested as severe pathological damage and functional impairment, and significant increase of Cr and BUN. After that, both pathological damage and functional parameter recovered gradually as reperfusion time extended (Fig. 1a–c). Meanwhile, we noticed that renal I/R-induced apoptosis of renal tubular epithelial cells with TUNEL assay was also increased obviously at 24 h after reperfusion, and the variation tendency of cell apoptosis was coincident with pathological damage and functional impairment (Fig. 1d). Increasing evidence suggests that ERS is involved in renal disease, continuous or excessive ERS is of great importance in apoptosis [27]. Thus, we determined changes of ERS-associated apoptosis pathways in renal tissues after I/R exposure. Results indicated that ERS related proteins, such as GRP78, XBP1 and CHOP in renal tissues, were all increased as reperfusion time extended and peaked at 16 or 24 h after reperfusion, which mirrored the patterns of pathological damage and functional impairment (Fig. 1e). These results indicated that ERS mediated apoptosis might be a potential mechanism contributing to I/R-induced AKI.

### Inhibition of ERS activation attenuated renal I/R-induced AKI

Figure 1 showed that expression of ERS-associated proteins, such as GRP78, XBP1and CHOP were significantly increased at 24 h after I/R in kidney tissues. In order to examine the role of ERS in I/R-induced renal injury, two well known ERS inhibitors, 4-PBA or TUDCA, were used to decrease ERS activation, and then, renal damage and cell apoptosis were determined.

**Fig. 2:**
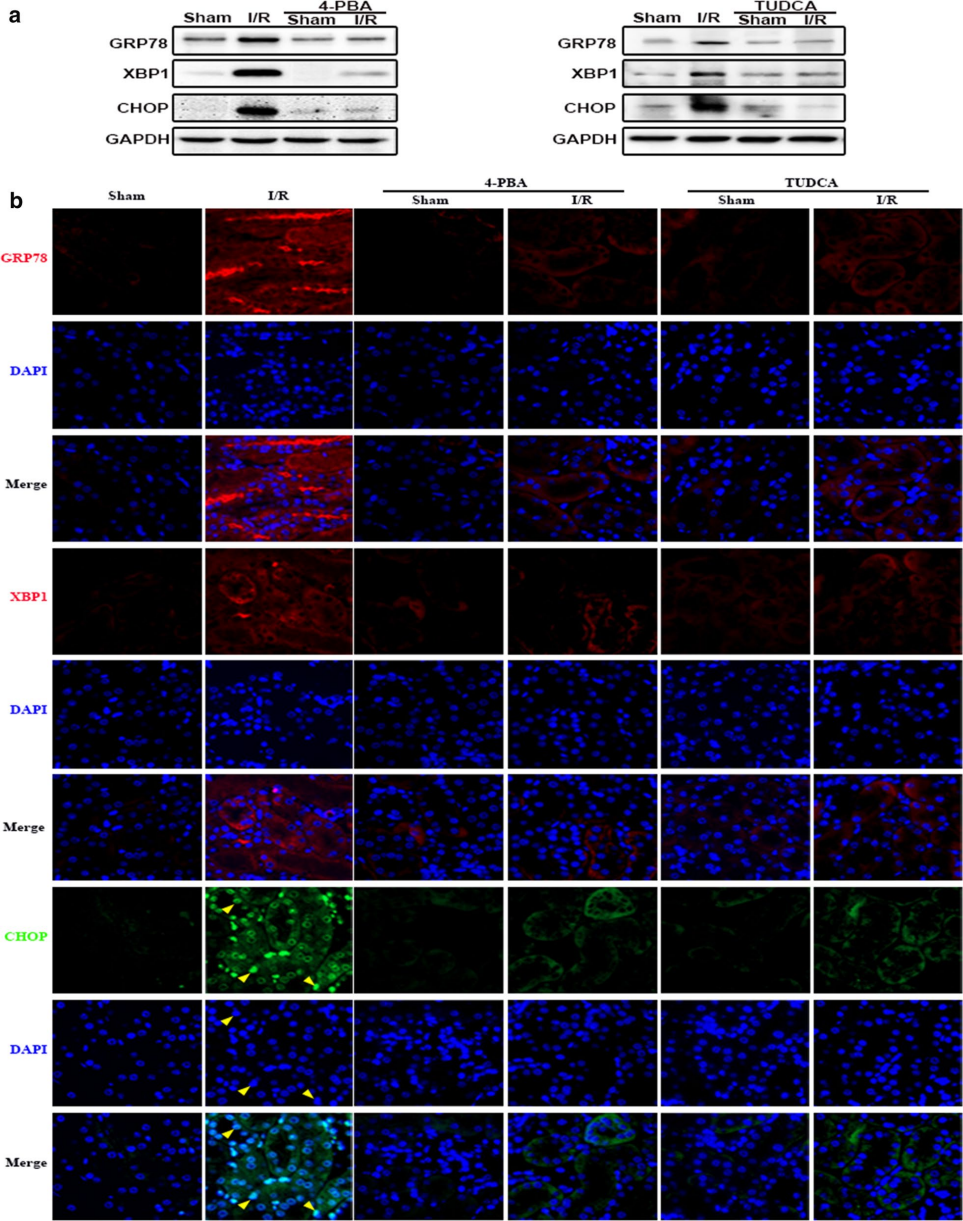
TUDCA and 4-PBA pretreatment attenuated I/R-induced ERS activation in renal tissues. TUDCA (300 mg/kg, 1 h) or 4-PBA (100 mg/kg, 1 h) pretreatment attenuated I/R (24 h)-induced GRP78, XBP1and CHOP expression increase in renal tissues. Changes of GRP78, XBP1and CHOP expressions were determined by **a** western blotting analysis. **b** Immunofluorescence staining (magnification ×400). Red: GRP78 and XBP1; green, CHOP; blue: the nuclear labeled by DAPI

In Fig. 2a, b, results obtained from western blotting or immunofluorescence demonstrated that both 4-PBA and TUDCA attenuated I/R-induced ERS activation effectively. GRP78, XBP1 and CHOP proteins were all localized on the cytoplasm of tubular epithelial cell before renal I/R, but alternations of their localization were different from each other when exposed to renal I/R injury. Figure 2b showed that renal I/R injury resulted in CHOP translocated from the cytoplasm to the nucleus, different from that GRP78 and XBP1 remained localizing on the cytoplasm of tubular cells (Fig. 2). The above data showed that 4-PBA or TUDCA pretreatment significantly attenuated I/R-induced ERS activation.

**Fig. 3:**
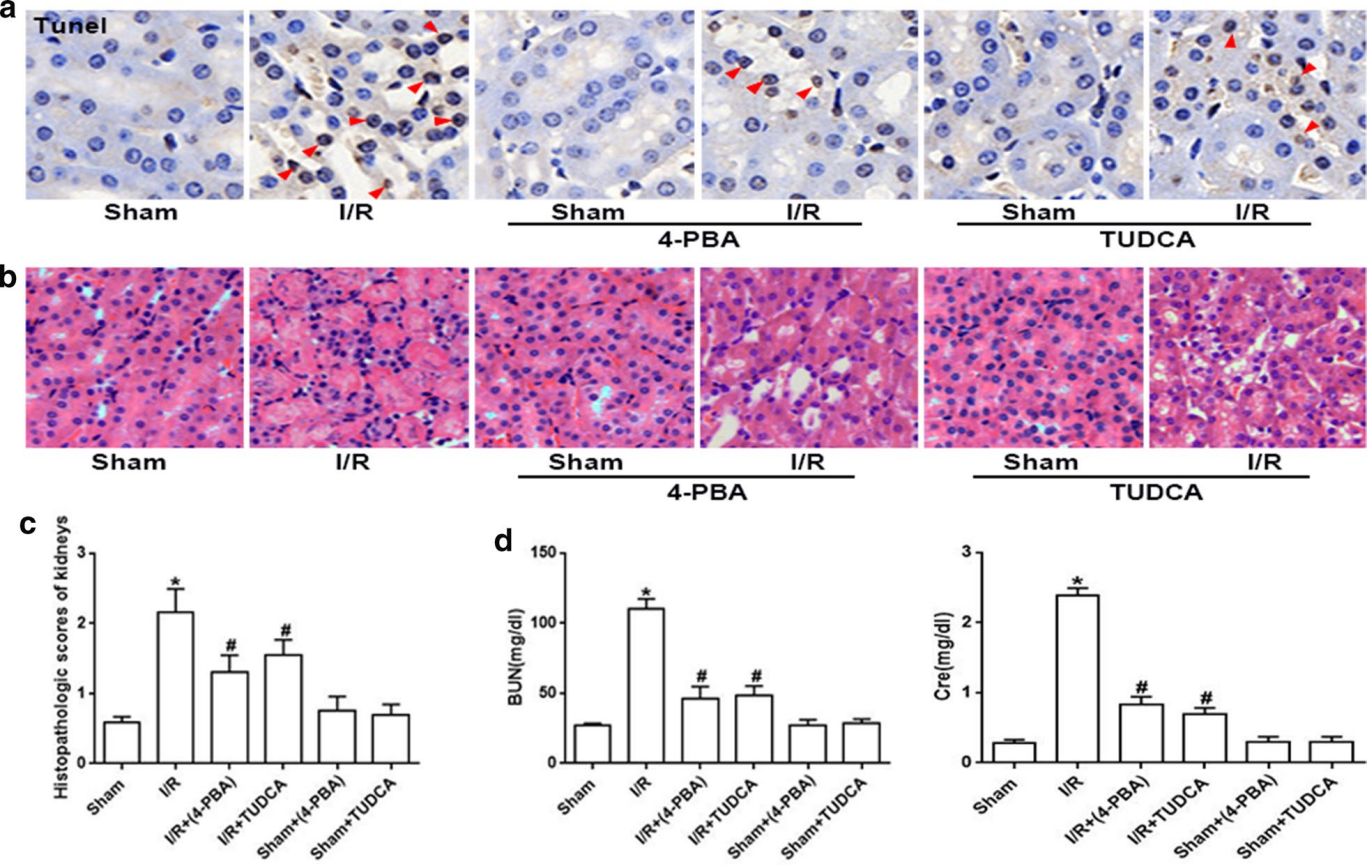
TUDCA and 4-PBA pretreatment attenuated I/R-induced renal tubular epithelial cells apoptosis and renal damage. Groups of mice were pretreated with 4-PBA (100 mg/kg, 1 h) or TUDCA (300 mg/kg, 1 h) and then were subjected to I/R (24 h) or Sham surgery. **a** Representative TUNEL apoptosis assay images of kidney tissues. Brown dots were deemed positive apoptosis cells (magnification ×400; red arrow). **b** Representative light microscopy images of H&E-stained sections from every group (magnification ×200). **c** Histopathological scores of kidneys, n = 6, *p < 0.05 vs Sham; ^#^p < 0.05 vs I/R group. Data are the mean values ± SEM. **d** Plasma BUN and Cre levels, n = 6, *p < 0.05 vs Sham; ^#^p < 0.05 vs I/R group. Data are the mean values ± SEM

More importantly, renal tubular epithelial cells apoptosis and renal damage induced by renal I/R were significantly attenuated as 4-PBA and TUDCA application, manifested as the reduction of TUNEL-positive cells (Fig. 3a), the improvement of pathological damage (Fig. 3b, c) and functional impairment (Fig. 3d). Therefore, we concluded that inhibition of ERS activation attenuated renal I/R-induced AKI.

### The elimination of ROS by NAC markedly alleviated ERS activation, as well as I/R-induced renal tubular epithelial cells apoptosis and tissue injury

Some researchers have pointed out that ROS might be a crucial inducer of the ERS response, which always contributed to cells death and tissues injury [28, 29]. Thus, we determined effects of ROS on ERS activation in renal I/R injury, as well as I/R-induced renal tubular epithelial cells apoptosis and renal tissue injury.

**Fig. 4:**
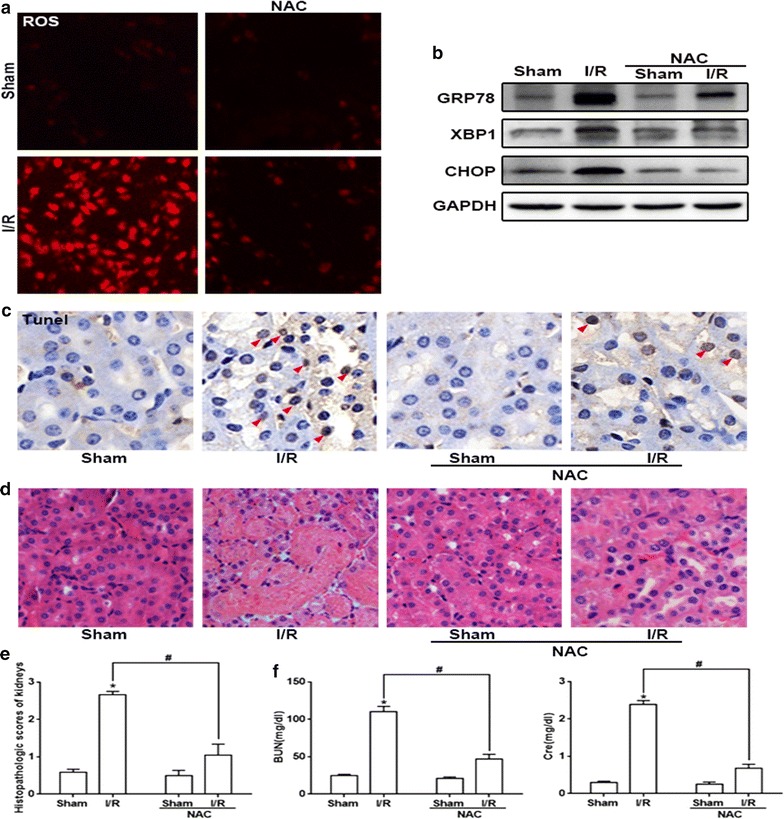
NAC pretreatment diminished ROS production, ERS-related proteins expression and apoptosis or damage. **a** NAC (200 mg/kg, 1 h) pretreatment reduced I/R (24 h)-induced ROS generation. DHE staining was used to detect the level of ROS (magnification ×200). **b** NAC pretreatment directly inhibited the I/R (24 h)-induced ERS-related proteins expression (GRP78, XBP1 and CHOP). **c** NAC pretreatment attenuated I/R (24 h)-induced apoptosis of kidneys. Brown dots were deemed positive apoptosis cells (magnification ×400; red arrow). **d**, **e** NAC pretreatment reduced renal injury (magnification ×200) and **f** decreased plasma BUN and Cre levels, n = 6, *p < 0.05 vs Sham group, ^#^p < 0.05 vs I/R group. Data are the mean values ± SEM

As expected, pretreatment of mice with NAC outstandingly neutralized the intracellular ROS generation in response to renal I/R treatment (Fig. 4a), which demonstrate the excellent anti-oxidation capability of NAC. Then, we implemented western blotting to evaluate whether NAC pretreatment inhibit the activation of ERS. Compared to the merely renal I/R group, NAC pretreatment greatly decreased the levels of GRP78, XBP1 and CHOP expression in kidneys (Fig. 4b), which demonstrated that renal ERS activation resulted by I/R could be attenuated by NAC application. And more importantly, as ROS was scavenged by NAC, renal tubular epithelial cells apoptosis and renal damage induced by renal I/R were attenuated significantly, manifested as the reduction of TUNEL-positive cells and the improvement of pathological damage and functional impairment (Fig. 4c–f). In summary, these data above proved that ROS elimination down-regulated ERS-mediated renal tubular epithelial cells apoptosis and protected against I/R-induced AKI.

### Cx32 gene knockout obviously decreased the level of ROS and ERS activation induced by I/R in renal tissues, which not only attenuated ERS-mediated cell apoptosis, but also improved renal damage

**Fig. 5:**
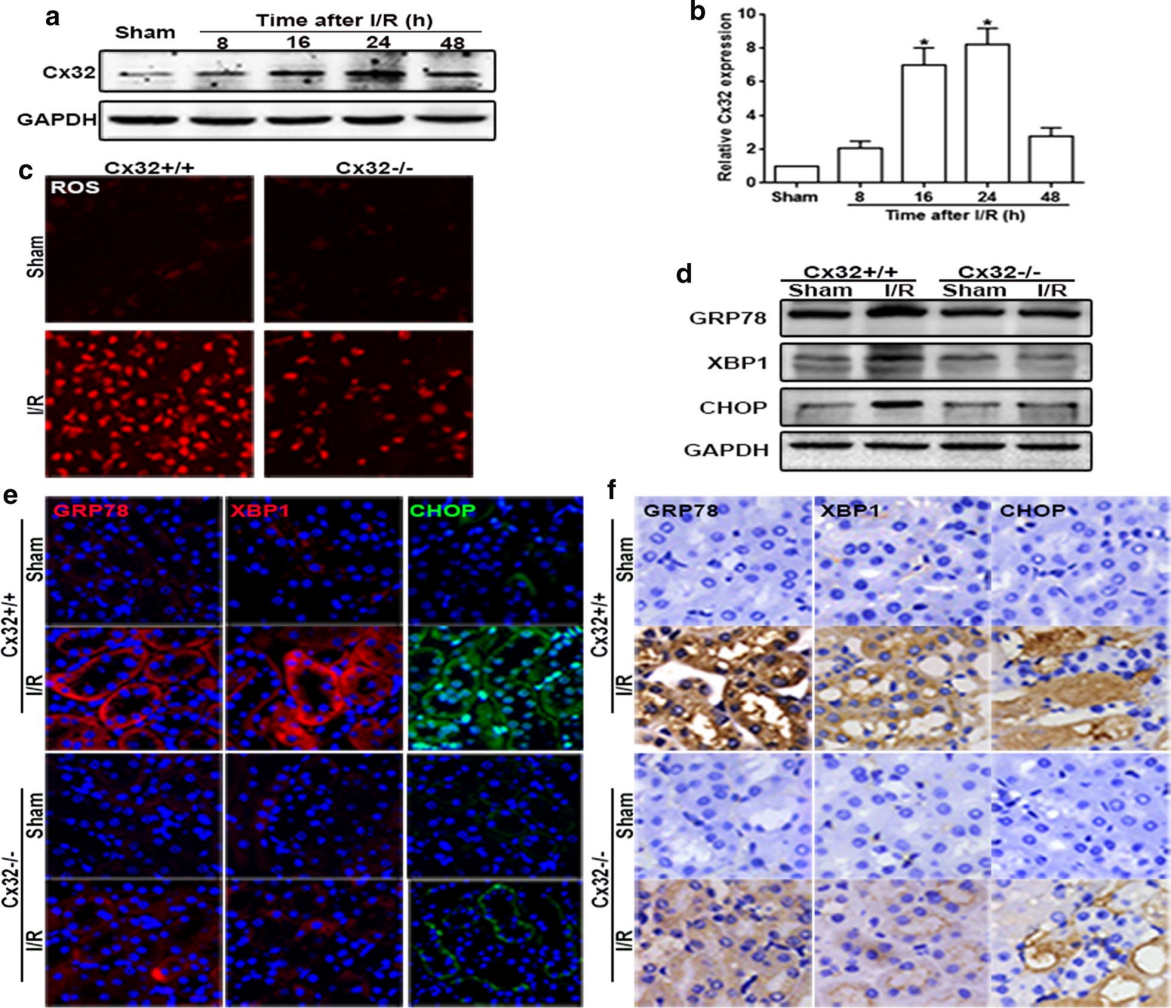
Cx32 gene deletion mitigated I/R-induced ROS generation and ERS activation. **a**, **b** Renal I/R induced the alteration of Cx32 protein expression was detected by western blotting analysis. n = 6, *p < 0.05 vs Sham group. **c** Cx32 gene deletion significantly alleviated ROS production and distribution (magnification ×200). **d**–**f** Cx32 gene deletion significantly alleviated I/R (24 h)-induced GRP78, XBP1and CHOP expression increase. Changes of GRP78, XBP1and CHOP expressions were determined by western blotting analysis. Immunofluorescence staining (magnification ×400; red: GRP78 and XBP1; green, CHOP; blue: the nuclear labeled by DAPI) and immunohistochemistry (magnification ×400)

**Fig. 6:**
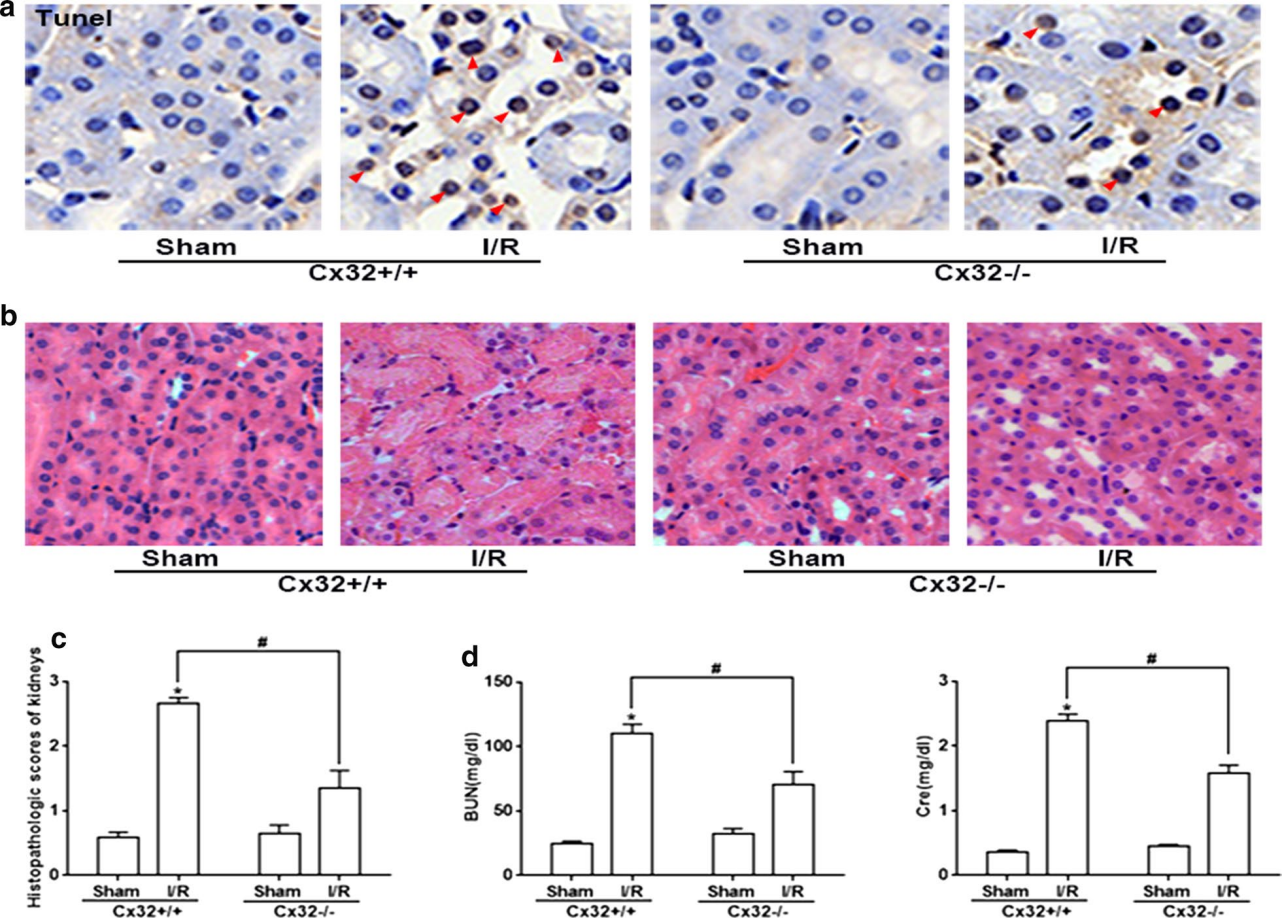
Cx32 gene deletion markedly attenuated I/R-induced renal tubular epithelial cells apoptosis and damage. **a** Cx32 gene deletion remarkably reduced I/R (24 h)-induced renal tubular epithelial cells apoptosis of kidneys. Representative TUNEL apoptosis assay images of kidney tissues. Brown dots were deemed positive apoptosis cells (magnification ×400; red arrow). **b** Histopathologic examination (magnification ×200). **c** Histopathologic scores of kidneys, n = 6, *p < 0.05 vs (Cx32^+/+^) +Sham group; ^#^p < 0.05 vs (Cx32^+/+^) + I/R group. Data are the mean values ± SEM. **d** Cx32 gene deletion significant decreased plasma BUN and Cre levels after renal I/R at the time point of 24 h, n = 6, *p < 0.05 vs (Cx32^+/+^) +Sham group; ^#^p < 0.05 vs (Cx32^+/+^) + I/R group. Data are the mean values ± SEM

Our previous study had demonstrated that Cx32 inhibition could protect against liver transplantation-induced AKI through mediating ROS distribution between the neighboring cells [6]. Thus, in our present study, we established renal I/R models with Cx32^+/+^ and Cx32^−/−^ mice to observe whether Cx32 could regulate ROS-associated ERS activation and its downstream renal tubular epithelial cells apoptosis and renal damage. Firstly, we found that the dynamic alteration of Cx32 expression in kidneys was coincident with I/R-induced renal damage and functional impairment: Cx32 expression peaked at 24 h after I/R, then gradually decreased (Fig. 5a, b), which suggested that changes of Cx32 expression might be closely related to I/R-induced renal damage. Next, we tested effects of I/R on the levels of ROS in renal tissues with or without Cx32 expression. Figure 5c showed that compared with Cx32^+/+^ mice, the increase of ROS in the renal tissue slice from Cx32^−/−^ mice was attenuated obviously after renal I/R, which further confirmed that Cx32 could regulate the levels of ROS in renal tissues. As the downregulation of ROS in Cx32^−/−^ mice, I/R-induced ERS activation was also decreased significantly, manifested as the reduction of GRP78, XBP1 and CHOP expression, and the decrease of CHOP nuclear translocation with three different methods: western blotting, immunohistochemical and immunofluorescence staining (Fig. 5d–f). We have concluded that ROS elimination or ERS activation inhibition could protect against renal tubular epithelial cells apoptosis and renal damage induced by renal I/R. And results in Fig. 5 demonstrated that Cx32 gene knockdown could attenuate ROS-induced ERS activation. Thus, we speculated that Cx32 gene knockdown could also reduce renal tubular epithelial cells apoptosis and renal damage. Figure 6 showed that our hypothesis was totally correct: after renal I/R, TUNEL-positive cells in the renal tissue slice from Cx32^−/−^ mice was attenuated obviously, compared to that from Cx32^+/+^ mice (Fig. 6a). Meanwhile, I/R-induced pathological damage and functional impairment was also improved significantly as Cx32 gene knockdown (Fig. 6b–d). These results suggested that Cx32 deficiency attenuated ROS-mediated ERS activation, which further improve renal I/R-induced AKI.

## Discussion

AKI is a life-threatening clinical emergency condition with a high case fatality and renal I/R is always considered to be an ordinary cause of AKI in humans [30, 31]. Although, various therapeutic strategies and agents targeted to ameliorate renal injury, the morbidity and death rate of renal I/R caused by AKI remains high. Until now, the explicit mechanisms responsible for the pathogeny of renal I/R injury were not fully understood, which lead to the lack of effective therapeutic strategies. Thus, in present study, we focused on the underlying mechanisms of I/R-induced AKI, in order to provide new and effective treatments. Our results demonstrated that renal damage is progressively exacerbated in a time-dependent manner at the reperfusion stage, which was consistent with the alternation of ERS activation, including GRP78, XBP1, and CHOP expression. Then, we found that 4-PBA or TUDCA, two well known ERS inhibitors, not only attenuated I/R-induced ERS activation effectively, but also improved renal tubular epithelial cells apoptosis and renal damage. We established renal I/R models with Cx32^−/−^ mice, and found that Cx32 deficiency attenuated ROS generation and distribution between the neighboring cells, which decreased I/R-induced ERS activation, and protected against cell apoptosis and renal damage. Thus, we concluded that Cx32 mediated ROS/ERS/apoptosis signal pathway activation played an important part in I/R-induced AKI.

Until now, about 21 isoforms of connexins had been found, which expressed in all human organs and tissues with distinct regulation and permeability [32, 33]. Different connexins could form GJs and regulate the transmission of intercellular signals between the neighboring cells, exerting different physiological and pathological roles [34]. Molecular signals, which enhanced cytotoxicity or induced apoptosis, were called “death signals”. The “death signals” transmitted through GJ could not only directly attack adjacent cells and lead to the cell death or apoptosis, but also could activate intracellular signaling pathways that were closely related to injury, and indirectly lead to the cell damage or apoptosis [6]. This kind of effect mediated by “death signals” is called “bystander effect”, causing cells or tissues damage amplification and deterioration [35, 36]. Cx32 is abundantly expressed in the renal tubular system, especially in proximal tubules, which plays a key role in maintaining and regulating the function of renal tubules. Recent studies showed that Cx32 expression always increased when liver or brain damage became more and more serious and these damages could be blocked by Cx32 inhibitors through decreasing oxidative stress and cell apoptosis [37, 38]. In present study, we found that Cx32 protein was increased and peaked at 24 h after renal I/R, which was coincident with the most severe kidney pathological damage and functional impairment (Figs. 1, 5). And we established renal I/R models with Cx32^−/−^ mice, and clarified that Cx32 deficiency could attenuate I/R-induced AKI specifically, which was coincident with our previous study that Cx32 inhibition protected against liver transplantation-induced AKI [6]. More importantly, Cx32 deficiency alleviated ROS transmitted between the neighboring cells (Fig. 5c) and inhibited ROS-related ERS activation, effectively (Fig. 5d–f), which was beneficial for protection against I/R-induced AKI. It can be seen that the renal Cx32 increase might be an important initiation factor for I/R-induced AKI, because of GJ composed of Cx32 mediating the “death signals” transmitting between the neighboring cells, which resulted in renal damage amplification and deterioration. And Cx32 deficiency or inhibition significantly depressed the transfer of “death signals”, protecting against renal damage amplification and deterioration. We believed that Cx32 might be a potentially effective therapeutic target for AKI treatment. Although we used Cx32^−/−^ mice to specifically observe effects of Cx32 on I/R-induced ROS and its downstream ERS activation and renal damage, factually, it was still puzzled that which affected the generation of oxidative stress, GJ composed of Cx32, Cx32 protein itself, or both? In order to clarify this problem, we observed effects of GJ composed of Cx32 on ROS thansfer in vitro (Additional file 1: Fig. S1). When GJ composed of Cx32 were inhibited by 2-APB (25 μM) on HK-2 cells (a kind of kidney tubular epithelial cells of human), ROS generation and distribution were both attenuated effectively. Our previous study had already demonstrated that 2-APB at the concentration of 25 μM just only inhibited function of GJ composed of Cx32, but had no effects on Cx32 expression itself [6]. These results indicated that GJ composed of Cx32 played an important part in oxidative stress in a Cx32-independent manner. GJ provides a fast pathway for the cell-to-cell transfer of small molecules. As reported, double-strand break formation mediated by Cx channels was detected in bystander cells as quickly as 2 min after irradiation [39]. While delayed changes such as cell cycle progression, changes in gene/protein levels of cell cycle regulatory proteins, which detected at later time points (i.e. several hours, days to weeks after radiation) were dependent on Cxs [39]. Therefore, we are more inclined to think that GJ composed of Cx32 mediating the transfer of “death signals” between the neighboring cells plays a greater role than Cx32 protein itself, especially in the early course of acute injury.

Although, “death signals” mediated by GJ had been investigated for a few years, the intrinsic essence of them had not been fully understood. Our previous results showed that ROS might be one of the most important “death signals”, because ROS was just one of the few signals that could be transmitted through GJ channels, and Cx32 channels inhibition could decrease ROS generation and distribution between the neighboring cells, which improved liver transplantation-induced AKI [6]. Normally, the levels of ROS in cells were very low, but played an important role in the regulation of physiologic tubular functions and renal microcirculation. When kidneys were exposed to I/R damage, intracellular ROS increased obviously which could be transmitted to the neighboring cells through GJ. The increase of ROS leads to the deterioration of the I/R-induced injuries. Thus, it could be seen that GJ mediated ROS transmission played an important role in the damage amplification caused by I/R, and both GJ function inhibition and ROS elimination might be effective strategies for renal protection. Our present study had proved this viewpoint and demonstrated that Cx32 deficiency or ROS scavenger could protect against I/R-induced renal damage.

When renal tissues or cells exposed to I/R damage, the content of ROS altered with the change of GJ function, but the underlying mechanism responsible to I/R-induced injuries was still not clear. Many researches had already showed that ROS played an essential role in decision of cell destiny: death or survival in some pathological process, including I/R injury [40, 41]. ROS could initiate the cascade of cell damage, apoptosis/necrosis, and pro-inflammatory responses, also could trigger cell apoptosis through various signaling pathways, such as inflammation, oxidative stress or ERS response [42, 43]. Recently, studies had shown that ROS destroyed ER function and initiated UPR and ERS in vivo and vitro, which might be an important mechanism that lead to tissues damage and cell apoptosis [44, 45].

ER was a chief organelle responsible for disposing proteins in the cell, perceiving injured cell and integrating apoptosis signals, then triggering cellular stress responses [15–17]. Various kinds of stresses, including I/R injury, could upset the ER homeostasis [46, 47]. To combat with various stresses, cell always activated a series of self-defense systems, such as upregulating ER chaperones and degrading misfolded proteins. But if the stress was overwhelming, ERS-initiated apoptotic cell death ensued [48]. In our present study, we found that in renal tissues subjected to I/R, ERS activation was significant, manifested as the increase of ERS-related protein, such as GRP78, XBP1 and CHOP. Meanwhile, 4-PBA and TUDCA application, usually acknowledged as ERS inhibitors and therapeutic agents to deal with ERS associated pathologies, effectively attenuated I/R-induced ERS activation (GRP78, XBP1 and CHOP expression were decreased and CHOP translocation from cytoplasm to nucleus was also attenuated), and more importantly, protected against renal tissues damage and cells apoptosis. What is more, we firstly demonstrated that Cx32 deficiency or ROS scavenger had the same effects as ERS inhibitors: not only inhibiting I/R-induced ERS activation, but also improving renal injuries and attenuating the number of apoptotic cells. Therefore, we believed that our research hypothesis was valid that Cx32 mediated ROS/ERS/apoptosis signal pathway activation was one of the most important mechanism of I/R-induced AKI.

In order to further confirm the conclusion, we supplement related experiments in vitro. Additional file 1 also showed that ROS increase caused by hypoxia 24 h and reoxygenation 4 h (H24R4) [6] in HK-2 cells (a kind of kidney tubular epithelial cells of human), could be reversed by 2-APB exposure effectively (a kind of specific inhibitor of Cx32 channels). What is more, 2-APB application attenuated H24R4-induced ERS activation (manifested as the downregulation of GRP78, XBP1 and CHOP expression) and simultaneously protected against H24R4-induced HK-2 cells damage. However, when HK-2 cells were preincubated with Tunicamycin (TM, a kind of ERS activator), protective effects caused by Cx32 channels inhibition were alleviated. These results complemented our research hypothesis that GJ composed of Cx32 mediated ROS/ERS activation-induced renal cell damage.

## Conclusions

Taken together, these findings totally substantiated our hypothesis that when exposed to I/R, the content of ROS in renal tissues was increased significantly, then transferred to the neighboring cells through GJ composed of Cx32, which lead to the activation of ERS in both local and remote renal tissues. This stress continuously and severely initiated ERS-related apoptotic signaling pathways, which accelerated renal cells apoptosis and gave rise to AKI ultimately. More importantly, our findings suggested that all of the strategies that Cx32 channels function decrease, ROS elimination, and ERS activation inhibition could attenuate I/R-induced renal cell apoptosis and AKI.

## Additional file

**Additional file 1.** In vitro studies, ERS activation could reverse protective effects of Cx32 channels inhibition on H24R4-induced HK-2 cells damage.

## Abbreviations

I/R: ischemia–reperfusion
AKI: acute kidney injury
ROS: reactive oxygen species
GJ: gap junction
Cx32: connexin32
ERS: endoplasmic reticulum stress
NAC: *N*-acetylcysteine
4-PBA: 4-phenylbutyric acid
TUDCA: tauroursodeoxycholic acid
UPR: unfolded protein response
BiP/GRP78: glucose regulated protein 78
ATF6: activated transcription factor 6
IRE1: inositol requiring enzyme
PERK: protein kinase-like ER kinase
XBP-1: X box-binding protein-1
CHOP: C/EBP homologous protein
PCR: polymerase chain reaction
H&E: hematoxylin and eosin
BUN: blood urea nitrogen
Cre: creatinine
DHE: dihydroethidium
2-APB: 2-aminoethoxydiphenyl borate
TM: tunicamycin
