# Correction to: Connexin32 plays a crucial role in ROS-mediated endoplasmic reticulum stress apoptosis signaling pathway in ischemia reperfusion-induced acute kidney injury

**DOI:** 10.1186/s12967-020-02375-z

**Published:** 2020-05-27

**Authors:** Yu Gu, Fei Huang, Yanling Wang, Chaojin Chen, Shan Wu, Shaoli Zhou, Ziqing Hei, Dongdong Yuan

**Affiliations:** Department of Anesthesiology, The Third Afliated Hospital of Sun Yatsen University, No. 600 Tianhe Road, Guangzhou, 510630 Guangdong Province China

## Correction to: J Transl Med (2018) 16:117 10.1186/s12967-018-1493-8

Following publication of the original article [1], the authors reported errors in Fig. 5a, e. The GAPDH band in Fig. 5a was duplicated with Fig. 4b GAPDH band. In Fig. 5e, the sham picture (Cx32+/+, sham, GRP78) and the sham pictures (Cx32−/−, sham, GRP78, CHOP) were duplicated. The correct version of Fig. 5 is given below.

**Fig. 5:**
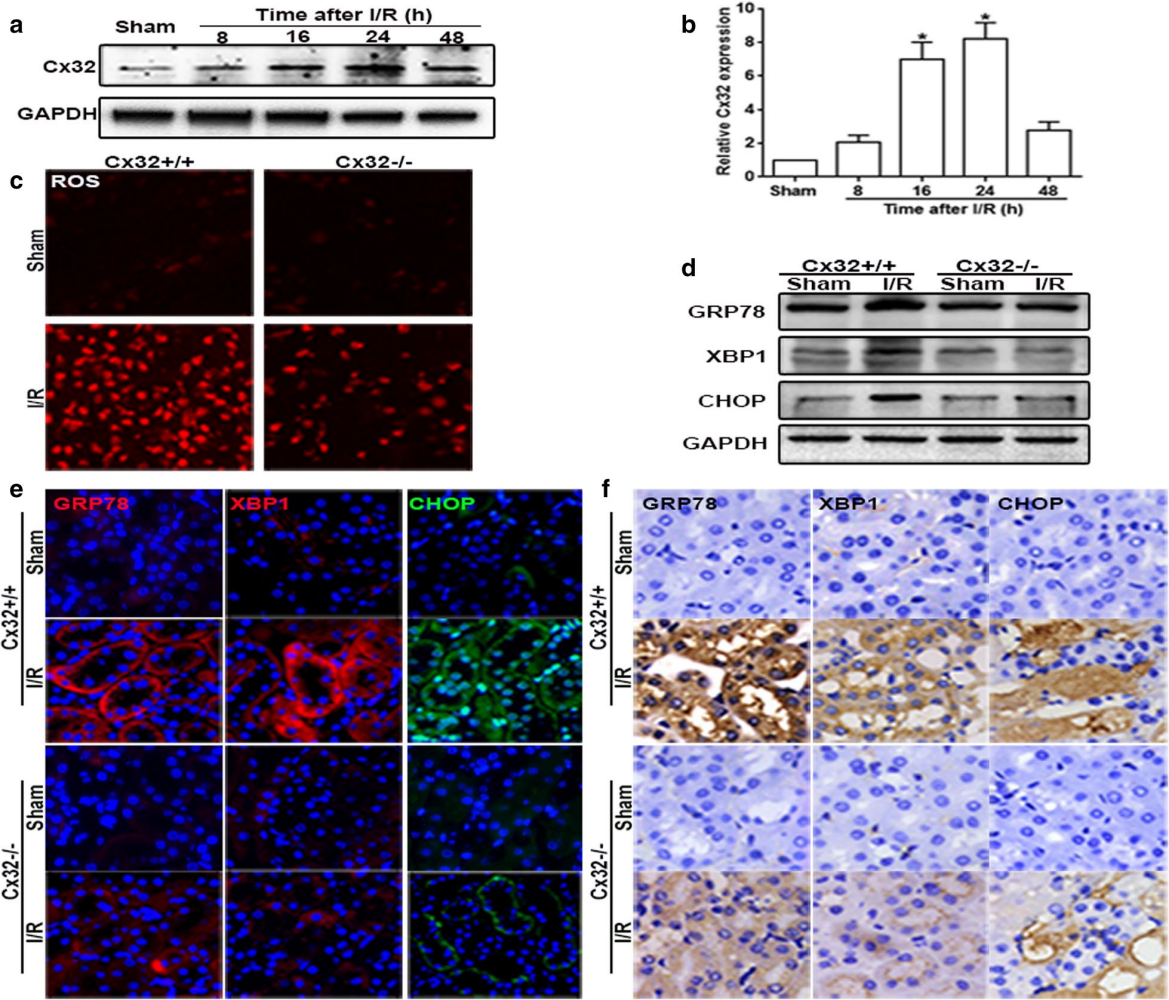
Cx32 gene deletion mitigated I/R-induced ROS generation and ERS activation. **a**, **b** Renal I/R induced the alteration of Cx32 protein expression was detected by western blotting analysis. n = 6, *p < 0.05 vs Sham group. **c** Cx32 gene deletion significantly alleviated ROS production and distribution (magnification ×200). **d**–**f** Cx32 gene deletion significantly alleviated I/R (24 h)-induced GRP78, XBP1and CHOP expression increase. Changes of GRP78, XBP1and CHOP expressions were determined by western blotting analysis. Immunofluorescence staining (magnification ×400; red: GRP78 and XBP1; green, CHOP; blue: the nuclear labeled by DAPI) and immunohistochemistry (magnification ×400)
