# Supplementary material for: Connexin32 plays a crucial role in ROS-mediated endoplasmic reticulum stress apoptosis signaling pathway in ischemia reperfusion-induced acute kidney injury
Source: J Transl Med. 2018 May 4;16:117. doi: 10.1186/s12967-018-1493-8 (PMC5935959; doi:10.1186/s12967-018-1493-8)
Supplement: Supplementary file 1 — Additional file 1. In vitro studies, ERS activation could reverse protective effects of Cx32 channels inhibition on H24R4-induced HK-2 cells damage. [file 12967_2018_1493_MOESM1_ESM.docx]

**Connexin32 plays a crucial role in ROS-mediated endoplasmic reticulum stress apoptosis signaling pathway in ischemia reperfusion-induced acute kidney injury.**

**Methods**

**Cell Culture and Treatment**

HK-2 cells (kidney tubular epithelial cells of human) were obtained from china center for type culture collection and cultured in DMEM containing 10% fetal bovine serum at 37℃ in a humidified atmosphere of 5% CO_2_ and 95% air. Cells hypoxia for 24h and reoxygenation for 4h (H24R4) model were performed as previously described [[1](#_ENREF_1)]. Briefly, cells were incubated in hypoxia incubator (5% CO_2_, 94% N_2_ and 1% O_2_) at 37°C for 24h, and then transferred back to a conventional incubator for 4h. Tunicamycin (TM, a kind of ERS activator, 0.01μM, 0.025μM, 0.05μM or 0.1μM) were used to induce ERS [[2](#_ENREF_2), [3](#_ENREF_3)]. 2-aminoethoxydiphenyl borate (a kind of specific inhibitor of Cx32 channels, 2-APB, 25μM) pretreated cells with or without TM (0.05μM) for 1h before inducing H24R4 injury.

**Cell viability assay**

HK-2 cells were seeded in 96-well plates. At the end of different stimulation, CCK-8 assay kit was used to [measure](javascript:;) the cells viability according to the manufacturer’s introduction.

**ROS detection in HK-2 cells**

HK-2 cells were seeded in 24-well plates with/without H24R4 treated. After different treatments, cells were washed with PBS and incubated in 2,7-dichlorodihydroflurescein diacetate (DCFH-DA, 10uM, Sigma-Aldrich) with serum free DMEM for 20 min at 37℃. Stained positive cells were observed under fluorescence microscope.

**Results**

**1. Inhibition of GJ composed of Cx32 attenuated ROS generation and distribution between the neighboring HK-2 cells, and protected against the H24R4-induced cell damage.**

When GJ composed of Cx32 were inhibited by 2-APB (25μM) on HK-2 cells (a kind of kidney tubular epithelial cells of human), H24R4-induced ROS generation and distribution were both attenuated effectively (Fig. S1A). Our previous study had already demonstrated that 2-APB at the concentration of 25μM just only inhibited function of GJ composed of Cx32, but had no effects on Cx32 expression itself. These results indicated that GJ composed of Cx32 played an important part in oxidative stress in a Cx32-independent manner. And simultaneously, with Cx32 channels inhibition and ROS reduction, H24R4-induced HK-2 cells damage was also decreased (Fig. S1B).


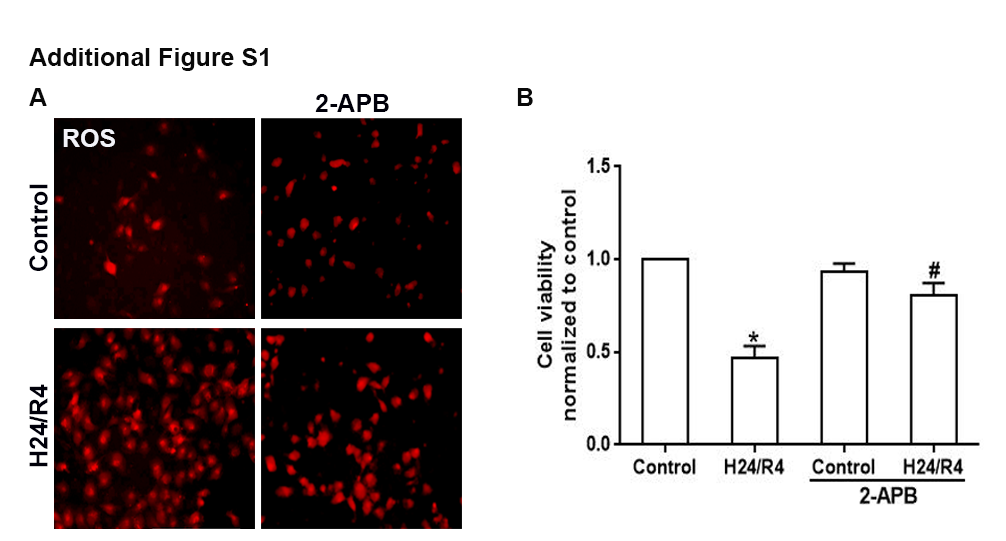


**Fig. S1** **Inhibition GJ composed of Cx32 with 2-APB** **attenuated ROS generation and distribution between the neighboring HK-2 cells, and protected against the H24R4-induced cell damage.**

(A) Effect of 2-APB on ROS generation and distribution between the neighboring HK-2 cells when exposed to H24R4, detected by DHE staining (magnification 200×). (B) Effects of 2-APB on HK-2 cells viability when exposed to H24R4, n=6, ^*^p<0.05 vs Control group; ^#^p<0.05 vs H24R4 group. Data are the mean values ± SEM. HK-2 cells were pretreated with 2-APB (25μM) 1h before H24R4.

**2. The ERS activator, TM application reversed protective effects of 2-APB on H24R4-induced ERS activation and HK-2 cells damage.**

We further clarified the causal relationship between Cx32 and ROS/ERS with TM, a kind of ERS activator in vitro. Fig. S2A showed that TM from 0.01 to

0.05μM had no significant effects on HK-2 cells viability.Fig. S2B demonstrated that Cx32 channels inhibtion with 2-APB attenuated H24R4-induced ERS activation (manifested as the downregulation of GRP78, XBP1 and CHOP expression), and simultaneously protected against H24R4-induced HK-2 cells damage (Fig. S1B and 2C). And even more, the protective effects caused by Cx32 channels inhibition could be alleviated by TM exposure, a classical activator of ERS (Fig. S1B and 2C). These results further confirmed that GJ composed of Cx32 could mediate H24R4-induced HK-2 cells damage through regulating ROS/ERS activation.


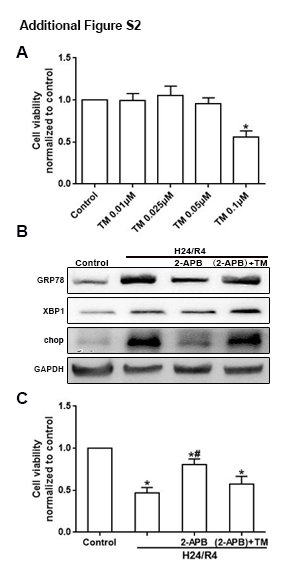


**Fig. S2 TM application reversed protective effects of 2-APB on H24R4-induced ERS activation and HK-2 cells damage.**

(A) Effects of TM (0.01μM, 0.025μM, 0.05μM or 0.1μM) on HK-2 cells viability in normal condition. n=6, ^*^p<0.05 vs Control group. Data are the mean values ± SEM. (B) Effects of TM and 2-APB on H24R4-induced ERS activation (including GRP78, XBP1 and CHOP expression) on HK-2 cells, examined with western blotting analysis. GAPDH was used as the loading control and for band density normalization. (C) Effects of TM and 2-APB on H24R4-induced HK-2 cells damage. n=6, ^*^p<0.05 vs Control group, ^#^p<0.05 vs H24R4 group. Data are the mean values ± SEM. HK-2 cells were pretreated with TM (0.05μM) or 2-APB (25μM) 1h before H24R4.

**References:**

1. Luo C, Yuan D, Li X, Yao W, Luo G, Chi X, Li H, Irwin MG, Xia Z, Hei Z: **Propofol attenuated acute kidney injury after orthotopic liver transplantation via inhibiting gap junction composed of connexin 32.** *Anesthesiology* 2015, **122:**72-86.

2. Patel SJ, Milwid JM, King KR, Bohr S, Iracheta-Vellve A, Li M, Vitalo A, Parekkadan B, Jindal R, Yarmush ML: **Gap junction inhibition prevents drug-induced liver toxicity and fulminant hepatic failure.** *Nat Biotechnol* 2012, **30:**179-183.

3. Yang Y, Cao MH, Wang Q, Yuan DD, Li L, Tao L: **The effects of 2-aminoethoxydiphenyl borate and diphenylboronic anhydride on gap junctions composed of Connexin43 in TM(4) sertoli cells.** *Biol Pharm Bull* 2011, **34:**1390-1397.
